# Development of a questionnaire for patient perception to functional appliances

**DOI:** 10.1186/s12903-024-04421-4

**Published:** 2024-06-08

**Authors:** Anosh A. Haik, Yassir A. Yassir

**Affiliations:** 1 Postgraduate student, College of Dentistry, University of Baghdad, Baghdad, Iraq; 2 Department of Orthodontics, College of Dentistry, University of Baghdad, Baghdad, Iraq; 3 School of Dentistry, University of Dundee, Dundee, UK

**Keywords:** Questionnaire, Patient perception, Functional appliances

## Abstract

**Objective:**

To developed and evaluate the validity, reliability, and Arabic translation of a questionnaire for preadolescent perception to removable functional appliances.

**Subjects and methods:**

A new questionnaire was drafted based on previously used questionnaires. Twelve orthodontic experts were selected within content and face validity panel and twenty preadolescents wearing functional appliances were participated in the face validation of the questionnaire. Two rounds of content validity were performed with the same experts. The final form of the validated questionnaire was translated from English to Arabic language. The content validity index (CVI) was used for content validity and the Cronbach’s alpha test was used to assess the internal consistency reliability of the questionnaire.

**Results:**

During the first round of content validity, 50 items were relevant to the underlying construct (Item-CVI ≥ 0.78), while four items were considered not valid (Item-CVI < 0.78) and the average scale-CVI was 0.93. In terms of face validation by experts, the percentage of agreement was adequate (96.4%). The questionnaire was modified by removing the non-valid items, adding/modifying items, and merging some categories. For second round of content validity, all items were found to be valid (I-CVI ≥ 0.78) and the overall questionnaire had adequate content validity (Scale-CVI/Ave = 0.94). The translated valid questionnaire also achieved a perfect agreement (100%) for face validity by patients. The internal consistency was appropriate (≥ 0.7).

**Conclusions:**

A new valid, reliable, and translated questionnaire (English and Arabic versions) that cover the majority of aspects of patients’ perception during treatment with removable functional appliances has been developed.

**Supplementary Information:**

The online version contains supplementary material available at 10.1186/s12903-024-04421-4.

## Introduction

Questionnaire is an instrument used for collecting information about individual’s observation, awareness and attitude in either written form (self-completed questionnaire) or interviews (face to face or telephone system). The planning to develop a questionnaire is a skill that requires cautious manipulative procedure and which should be approved and validated by experts [1, 2].

Questionnaire validation ensures the ability of the scale to achieve its objective for assessment the durability of collected data that influenced by a lot of factors which are difficult to be controlled. The validated questionnaire means it could measure what it was designed to measure and should be understood easily to be answered in a correct way [3, 4]. There are many divisions and subdivisions of validation procedures and each questionnaire can be validated with only one type not the others [5]. Content validity is a quantitative test to check the content of the questionnaire is fully addressed, items are relevant to the underlying context and to eliminate irrelevant ones by using a Content Validity Index (CVI). This procedure is performed only by a panel of at least three specialist judgments (more experts is more valuable) to determine the relevancy of the items. Usually, using two rounds of validation in necessary after a period of time not less than 10 days from first round to refine any amendment [6–8]. On the other hand, face validation is the qualitative method to measure the representativeness of the content of the questionnaire on the face of it in form the of clarity of language, readability, feasibility, and formatting consistency of questionnaire. Face validation is the weakest and simplest type of validation and sometimes be confused with content validation as they flow in the same stream, yet it is simple and can be performed by both experts and participants [9–11]. To determine if the questionnaire is measuring what was intended to measure, it is necessary to include both face and content validation so this approach is known as “validation by assumption” [12].

To make the questionnaire applicable for cross-cultural research, translation may be required to allow cultural adaptation and giving an advantage of making universal evaluations. The translation procedure is a big challenge process as the items of the translated version should maintain the meaning and intent of the original ones. There are different methods for translation; translation by unqualified translator without validation of translated version (simplest method), translation by qualified team (more than one), or back-translation method (more preferable and expensive method) [2, 13, 14].

Eventually, it is important to review the degree of agreement between the questionnaire contents either by administrating the questionnaire with the same observer in different occasions (Intra-examiner Reliability) or with different observers (Inter-examiner Reliability). Adequate questionnaire reliability enhances the correlation between items (internal consistency) and the reproducibility of questionnaire (stability) [5, 15, 16].

Nowadays; individual perception for their quality of life (QoL) is of great importance during oral health care. Oral health studies focus on this aspect and formulate the Oral Health Related Quality of Life (OHRQoL). The scales of OHRQoL are usually concentrated on patients need for treatment to improve their QoL with little attentions to the patients’ perception during treatment. In orthodontic field and for effective orthodontic appliance; technically the appliance must be functional and effective; practically it must be easily used, and comfortable for their users to enhance its success. So that including patients’ perceptions for new orthodontic treatment and appliance will provide researchers a view about the pros and cons of the treatment through assessing different aspects of OHRQoL [16–18]. Currently, new designs of orthodontic functional appliance have been developed using different materials. Therefore, assessing the impact of these appliances during treatment is imperative. This study was designed to develop and validate a questionnaire for patients with functional appliances, which has been based on several questionnaires [19–26]. The study will also include Arabic translation and assessing the reliability of the questionnaire.

## Subjects and methods

### Study design

This is a cross sectional study which was designed as a part of randomized clinical trial. Ethical approval was obtained from the ethics committee at the Collage of Dentistry, University of Baghdad (Reference No. 664 in 13.9.2022).

### Design the questionnaire

A pool of questions from different questionnaires investigating several domains about patient experience during functional orthodontic treatment were implemented to be used in this questionnaire in its initial draft [19–26].

### Sample

The target group for questionnaire validation consists of:

- A quota sample of 12 experts (orthodontic specialists) was selected to validate this questionnaire content and face validation). They worked in educational and health sectors with different levels of experience (more than 10 years).
- Twenty preadolescent patients were selected from governmental specialized dental centers, private clinic, and from Department of Orthodontics at the Collage of Dentistry- University of Baghdad. All were wearing functional appliances after 4–6 months from their treatment. Those patients participated in the face validation of the questionnaire.

### Validation Procedure

To develop a relevant, validated, and understandable questions for Arabic-speaking patients, the initially selected questions were passed through the following steps:

#### Step 1: first round of content and face validation by experts

Each specialist received an invitation letter to participate in a content validity panel and asked to rate each item in the questionnaire independently using a 4-point Likert scale (score 1 = not relevant, score 2 = somewhat relevant, score 3 = relevant, and score 4 = very relevant). Accordingly, any item scored as 1 or 2 was considered as not relevant, while items scored as 3 or 4 means they were relevant. The quantitative method by content validity index (CVI) was used to assess the items/questionnaire. According to Lynn’s method; item level-CVI (I-CVI) was calculated by diving numbers of experts who rated each item as score 3 or 4 to the total number of experts. The accepted level of each item in order to be valid must be I-CVI ≥ 0.78. Since the total number of expert raters in this study was 12, at least 10 experts should score the item with 3 or 4 to be retained, otherwise the item should be considered not valid. For assessing the validity of the overall questionnaire, the scale level of CVI (S-CVI) was calculated also by averaging the I-CVI for all items. The recommended and accepted level of S-CVI is 0.9 [6, 27].

The same experts who participated in the content validity panel were also received an invitation letter to be a part of the face validity panel to qualitatively assess the appropriateness and readability of the questionnaire through eight questions and an open-end question to add their feedbacks and suggestions to modify the questionnaire. This process was performed using a 4-point Likert scale (score 1 = strongly disagree, score 2 = disagree, score 3 = agree, and score 4 = strongly agree).

#### Step 2: questionnaire modification

According to the result of the first step, the questionnaire was amended by modifying/merging some items, adding other items, and excluding the non-valid items (I-CVI < 0.78).

#### Step 3: second round of content validation by experts

The same 12 experts were also invited for the second round of content validation to assess the modified version of the questionnaire.

#### Step 4: translation process

The final form of the validated questionnaire was then translated from English to Arabic language via an official bureau. The translation process was performed by a qualified and professional team which consisted form three translators who were fluent in Arabic and English languages. Two translators transformed the English form of questionnaire to Arabic form independently then the team leader reviewed and evaluated the two versions to refine the language and produce a single Arabic version.

#### Step 5: face validation by patients

Twenty patients who were participated in the randomized clinical trial with myofunctional appliance treatment were asked to participate in this step of face validation. They were provided with a copy of the validated and translated questionnaire and asked to assess the readability and easiness to understand the items of the questionnaire via a face validation form.

#### Step 6: reliability

The validated and translated questionnaire was evaluated for internal consistency reliability (Cronbach’s alpha test) to measure the correlation between the items of the questionnaire. As this questionnaire determined the experience of orthodontic patients during treatment with functional appliances so the situation and response of patient to treatment would change with time making the possibility of measuring the repeatability (test-retest) difficult [22].

### Statistical analysis

Statistical Package for Social Science version 25.0 (SPSS Inc., Chicago, IL. **USA)** was used for statistical analysis with statistical significance was set at *P* < 0.05.

All responses (first and second round content validation, face validation forms) were collected through direct contact and were saved as an Excel spread sheet (Excel, Microsoft Office Professional Plus 2019, Washington, USA). Content validity was calculated by I-CVI and S-CVI values, while face validation was assessed according to the percentage of satisfactory agreement scores for each question within the form. Cronbach’s alpha test was performed to measure the internal consistency reliability.

## Results

A total 12 orthodontic experts and 20 preadolescent patients were recruited into this part of study and feedback was obtained from them to develop the final form of validated questionnaire for patient perception to functional appliances throughout the following steps:

### Step 1: first round of content and face validation by experts

Regarding this round of content validity, 50 items were relevant to the underlying construct (I-CVI ≥ 0.78), while 4 items were considered not valid (I-CVI < 0.78). Therefore, the questionnaire was revised by removing the four non-valid items. The S-CVI for the overall questionnaire (average) was 0.93, which is above the threshold (0.90) for questionnaire validity (Table 1). In terms of face validation, the questionnaire was considered adequate with overall agreement of 96.4 per cent. Some experts suggested modifications for certain items to be more appropriate.

**Table 1:** First round of content validation by 12 orthodontic experts

Category	No.	Questionnaire Items	I-CVI	Validity
*Your experience of wearing your appliance*	1	Does wearing the appliance as what you expected?	0.83	Valid
	2	Has the appliance ever broken during treatment time?	1.00	Valid
	3	Have you had any extra appointments because your appliance was broken or not fitting well?	1.00	Valid
	4	If you had extra appointments because your appliance was broken or not fitting well, has this bothered you?	0.83	Valid
	5	It was hard to keep the appliance clean	0.92	Valid
	6	Does the appliance fall out during sleep?	1.00	Valid
	7	How do you find wearing and removing the appliance?	1.00	Valid
	8	Do you experience any gag reflex while wearing the appliance?	1.00	Valid
*How have the following things changed due to wearing your appliance?*	9	Speech	0.92	Valid
	10	Eating	0.83	Valid
	11	Drinking	0.83	Valid
	12	Breathing	1.00	Valid
	13	Sleeping	1.00	Valid
	14	Studying	0.83	Valid
	15	Appearance	1.00	Valid
	16	If you were teased or bullied about your teeth before you started treatment, has this changed?	1.00	Valid
*How have the following affected you due to wearing your appliance?*	17	Pain in your teeth	1.00	Valid
	18	Pain in your mouth	0.83	Valid
	19	Pain in your jaw or temporomandibular joints	1.00	Valid
	20	Pain from rubbing	0.67	Non-valid
	21	If there was pain, did you use medications to relieve it?	1.00	Valid
	22	Feeling embarrassed	1.00	Valid
	23	Dribbling (Uncontrolled salivation)	1.00	Valid
*School work / activity*	24	How has your school work / activity been affected due to wearing your appliance?	1.00	Valid
	25	Appearance	1.00	Valid
	26	Speech	0.92	Valid
	27	Eating	0.83	Valid
	28	Studying	0.92	Valid
	29	Pain in your teeth	0.92	Valid
	30	Pain in your mouth or jaw	0.92	Valid
	31	Feeling embarrassed	1.00	Valid
	32	Drilling (Uncontrolled salivation)	1.00	Valid
	33	Teasing or bullying	1.00	Valid
*Social relationships*	34	How have your interaction with friends and family been affected due to wearing your appliance?	1.00	Valid
	35	Appearance	1.00	Valid
	36	Speech	0.92	Valid
	37	Eating	0.83	Valid
	38	Studying	0.75	Non-valid
	39	Pain in your teeth	0.83	Valid
	40	Pain in your mouth or jaw	0.83	Valid
	41	Feeling embarrassed	1.00	Valid
	42	Drilling (Uncontrolled salivation)	0.92	Valid
	43	Teasing or bullying	1.00	Valid
*Hobbies / Interests*	44	If you are practicing any hobby, how have this been affected due to wearing your appliance?	0.88	Valid
	45	Appearance	1.00	Valid
	46	Speech	0.92	Valid
	47	Eating	0.75	Non-valid
	48	Studying	0.58	Non-valid
	49	Pain in your teeth	0.92	Valid
	50	Pain in your mouth or jaw	0.92	Valid
	51	Feeling embarrassed	1.00	Valid
	52	Drilling (Uncontrolled salivation)	0.92	Valid
	53	Teasing or bullying	1.00	Valid
*Your advice to other patients*	54	Based upon YOUR experience, would you recommend your appliance to someone who has similar malocclusion?	1.00	Valid
*S-CVI/Ave*			**0.93**	

### Step 2: questionnaire modification

The questionnaire was modified according to the experts’ suggestions as follows:

- Removing four non-valid items (items 20, 38, 47, and 48).
- Merging seven main categories to five.
- Adding two items in the second and third categories, namely: (‘Brushing and maintaining oral health’, ‘If there was pain, did this affect wearing of appliance?’).
- Modifying two items (question 18 was changed (from ‘Pain in your mouth’ to ‘Pain or ulceration in your mouth (due to pressure)’ while question 19 was changed from ‘Pain in your jaw or temporomandibular joints’ to ‘Pain/Clicking in your jaw or temporomandibular joints’.

The new draft of the validated questionnaire included 37 questions within five categories (Supplementary Table 1).

### Step 3: second round of content validation by experts

During this step, a second round of validation was implemented for the two added and two modified items of questionnaires by the same 12 orthodontic experts after two weeks. The four items were found to be valid (I-CVI ≥ 0.78) and the overall questionnaire had almost perfect content validity (S-CVI/Ave = 0.94) (Table 2).

**Table 2:** Second round of content validation by 12 orthodontic experts

Category	Added\Modified items	Questionnaire Items	I-CVI	Validity
**How have the following things changed due to wearing your appliance?**	Added	Brushing and maintaining oral health	1.00	Valid
**How have the following affected you due to wearing your appliance?**	Modified	Pain or ulceration in your mouth (due to pressure)	1.00	Valid
	Modified	Pain/clicking in your jaw or temporomandibular joints	0.83	Valid
	Added	If there was pain, did this affect wearing of appliance?	0.92	Valid
**S-CVI/Ave**			**0.94**	

### Step 4: translation process

The final and new form of the validated questionnaire was translated from English to Arabic language via an official bureau. This form was also checked by the authors to ensure that the translation did not change the main idea of the items (Supplementary Table 2).

### Step 5: face validation by patients

Twenty preadolescent patients reviewed the questionnaire, and their feedback stated that it had clearly and untestable items with consistent format and style. The questionnaire achieved a perfect agreement for face validity by patients (overall agreement = 100%).

### Step 6: reliability

According to the nature of responses, the questionnaire was divided into two sections in order to be tested with the Cronbach’s alpha coefficient test. The first section included two domains: “Your experience of wearing your appliance” and “How have the following affected you due to wearing your appliance?”. The second section also included two domains: “How have the following things changed due to wearing your appliance?” and “How wearing the appliance can affect other things in your life” (school work/activity, social relationships, hobbies/interests). The first section achieved Cronbach’s alpha level of 0.714 and the second Sect. 0.823.

## Discussion

Individuals have suffered from various degree of malocclusions and suffering more during the course of orthodontic treatment with different types of appliances. These complaints affect their QoL, especially when the target population are in stage of physical, intellectual, psychological and social challenges (preadolescence) dealing with bulky and removable orthodontic appliances [28, 29]. This study was conducted to provide a valid and translated questionnaire to evaluate the impact of functional appliances on Arabic-speaking preadolescents during treatment.

### Design the questionnaire

It was instituted that the target group of a questionnaire is an important part during designing the questionnaire [18]. The sample that filled the questionnaire in this study were preadolescents having the same skeletal malocclusion (skeletal class II), Since this age group is usually undergo emotional changes, moreover their social relations during activities are important, therefore, the initial draft of study questionnaire was designed to accommodate these experiences after 4–6 months from using removable functional treatment.

The English form of the questionnaire in the present study is based on several other questionnaires with certain modification to be suitable for existing study purpose. This was a common procedure in other studies, such as Bos et al. [30] and Yassir et al. [22]. who designed and modified a questionnaire for fixed orthodontic appliances based on questionnaires of other type of appliances. Other studies [19–21, 24–26] used a questionnaire that focused to express patient experience to removable or fixed functional appliances, while Golfeshan et al. [23]. focused only on patient satisfaction. Those studies concerned on one aspect and were not being comprehensive to include further aspects that could affect teenagers QoL in school, during hobbies and their relations with family and friends. Consequently, the present questionnaire was developed to cover the majority of aspects that interfere with the preadolescents’ QoL and specified for removable functional appliances.

### Content and face validation by experts

Validity of a questionnaire is the key factor for evaluating the precision, accuracy and ability to understand the developing instrument in a correct and easy way [4, 31]. To validate any questionnaire, it is necessary to include both qualitative and quantitative methods of validation as reveled by previous study [22].

Increasing the number of experts could enhance the validity procedure. This will provide an excellent opportunity to collect more opinions and ratings, increase strength of certain items rating and reduce rating of others because of inter-professional differences (different insights to specific situation based on different levels of education and experiences) [6, 31, 32]. In the present study, 12 orthodontic specialists form different workplaces with adequate clinical and teaching experiences (15–26 years of experiences) were participated in both content and face validation following the guidelines of Grant and Davis [33] and Rubio et al. [34]. .

During the first round of content validation, 50 items from a total of 54 were valid and relevant to the underlying construct (I-CVI ≥ 0.78), while 4 items were considered not valid (I-CVI < 0.78). The S-CVI for the overall questionnaire was 0.93, which is above the threshold (0.90) for questionnaire validity. Comparing these results with the score levels revealed by Lynn [6] and Polit et al. [8]. , the content validity is excellent and the questionnaires required little modifications which included removing the non-valid items.

Clarity of language and readability were assessed during the face validation. Furthermore, any feedback from the expert to modify the questionnaire was also feasible during this stage. This in turn was emphasized and considered an important step to modify any questionnaire [6, 11, 35]. The results were considered adequate with overall agreement of 96.4 per cent. The feedback included adding, merging, and modifying some items in order to cover some missing information and make the questionnaire shorter.

Haynes et al. [36]. and Rubio et al. [34]. stressed on the importance for the second round of validation. Lynn [6] recommended ten days at least as a time for a second round of validation by the same experts. For that reason, the same 12 experts invited again after about two weeks for a second round of content validation for the modified items. All items were rated as relevant (valid) and the S-CVI for the overall questionnaire was 0.94.

### Translation process

During this step, the final form of the validated questionnaire was translated from English to Arabic language via an official Bureau for translation by a qualified and professional team. The team consisted form three translators who were fluent in Arabic and English languages. Two translators transformed the English form of questionnaire to Arabic independently, then the team leader checked and evaluated the two versions to refine the language and produce a single Arabic version. This procedure was in accordance with the guidelines provided by Göranson et al. [14] who confirmed that one of the available standard methods for translation is by a qualified team (more than one translator). The original meaning of each item was also checked in order not to be changed (by the team) using a back translation method according to guidelines for standard translation by WHODAS2.0 [37].

### Face validation by patients

Twenty patients were provided with a copy of the validated and translated questionnaire and were asked them to assess the readability and easiness to understand the items of the questionnaire via a *face validation form* following the strategies of Zamanzadeh et al. [10], Yassir et al. [22]. The questionnaire achieved a perfect agreement for face validity by patients. the language was easily readable and none of respondents needed help to complete the questionnaire.

### Reliability

The questionnaire showed acceptable and good internal consistency as revealed by the scores of Cronbach’s alpha coefficient test for first and second Sect. (0.714 and 0.823 respectively) signifying that the scale was reliable and homogenous. As this scale measured a changeable condition over time, so test-retest reliability is not applicable to measure the stability of the scale [15].

### Strength and limitation of the study

The strengths of the study:

- The responded sample were selected from governmental specialized dental centers, private clinics, and from the Department of Orthodontics at the Collage of Dentistry-University of Baghdad which could enhance the generalizability of the outcomes.
- The clinical work of the study was performed in single dental clinic with single clinician to reduce the possibility of performance bias.
- The questionnaire was filled by the participants without any interference from their parents or guardian assistance.

The limitations of the study:

- This valid questionnaire is only suitable for assessing patient perception during the progress of the treatment.
- Test-retest stability of the questionnaire cannot be measured for this changeable condition.
- Construct validity is required to complete the validation of this questionnaire. However, this was not possible due to small sample size. This could be considered as new research.

## Conclusions and suggestions

- A new valid, reliable, and translated questionnaire (English and Arabic versions) that cover the majority of aspects of patients’ perception during treatment with removable functional appliances has been developed.
- Applying the questionnaire in different Arabic countries is suggested to assess a cross-cultural variation and validity of the new questionnaire.

### Electronic supplementary material

Below is the link to the electronic supplementary material.

Supplementary Material 1

## Data Availability

The datasets used and/or analysed during the current study available from the corresponding author on reasonable request.
