# Supplementary material for: Development of a questionnaire for patient perception to functional appliances
Source: BMC Oral Health. 2024 Jun 8;24:675. doi: 10.1186/s12903-024-04421-4 (PMC11161958; doi:10.1186/s12903-024-04421-4)
Supplement: Supplementary file 1 — Supplementary Material 1 [file 12903_2024_4421_MOESM1_ESM.docx]

**Development of a questionnaire for patient perception to functional appliances**

Supplementary Table 1: English form of a validated questionnaire

**Your experience of wearing your appliance**

| Does wearing the appliance as what you expected? | No | Not sure | Yes |
| --- | --- | --- | --- |
| Has the appliance ever broken during treatment time? | Not at all | A little | A lot |
| Have you had any extra appointments because your appliance was broken or not fitting well? | No |  | Yes |
| If you had extra appointments because your appliance was broken or not fitting well, has this bothered you? | Not at all | A little | A lot |
| It was hard to keep the appliance clean | Not at all | A little | A lot |
| Does the appliance fall out during sleep? | Not at all | A little | A lot |
| How do you find wearing and removing the appliance? | Easy |  | Difficult |
| Do you experience any gag reflex while wearing the appliance? | Not at all | A little | A lot |

**How have the following things changed due to wearing your** **appliance?**

| Speech | Improved | No change | Worse |
| --- | --- | --- | --- |
| Eating | Improved | No change | Worse |
| Drinking | Improved | No change | Worse |
| Brushing and maintaining oral health | Improved | No change | Worse |
| Breathing | Improved | No change | Worse |
| Sleeping | Improved | No change | Worse |
| Studying | Improved | No change | Worse |
| Appearance | Improved | No change | Worse |
| If you were teased or bullied about your teeth before you started treatment, has this changed? | Less | No change | More |

**How have the following affected you due to wearing your appliance?**

| Pain in your teeth | Not at all | A little | A lot |
| --- | --- | --- | --- |
| Pain or ulceration in your mouth (due to pressure) | Not at all | A little | A lot |
| Pain/clicking in your jaw or temporomandibular joints | Not at all | A little | A lot |
| If there was pain, did you use medications to relieve it? | Not at all | A little | A lot |
| If there was pain, did this affect wearing of appliance? | Not at all | A little | A lot |
| Feeling embarrassed | Not at all | A little | A lot |
| Dribbling (Uncontrolled salivation) | Not at all | A little | A lot |

**We would like to know if wearing the appliance can affect other things in your life**

**SCHOOL WORK / ACTIVITY**

**How have your school work / activity been affected due to wearing your appliance?**

| Improved | No change | Worse |
| --- | --- | --- |

**GETTING ON WITH FRIENDS AND FAMILY (SOCIAL RELATIONSHIPS)**

**How have your interaction with friends and family been affected due to wearing your appliance?**

| Improved | No change | Worse |
| --- | --- | --- |

**HOBBIES / INTERESTS**

**If you are practicing any hobby, how have this been affected due to wearing your appliance?**

| Improved | No change | Worse |
| --- | --- | --- |

**If Improved or Worse, this was due to**

| Appearance | Improved | Worse | - School/Work - Social Relationship - Interests |
| --- | --- | --- | --- |
| Speech | Improved | Worse | - School/Work - Social Relationship - Interests |
| Eating | Improved | Worse | - School/Work - Social Relationship |
| Studying | Improved | Worse | - School/Work |
| Pain in your teeth | Improved | Worse | - School/Work - Social Relationship - Interests |
| Pain in your mouth or jaw | Improved | Worse | - School/Work - Social Relationship - Interests |
| Feeling embarrassed | Improved | Worse | - School/Work - Social Relationship - Interests |
| Dribbling (Uncontrolled salivation) | Improved | Worse | - School/Work - Social Relationship - Interests |
| Teasing or bullying | Improved | Worse | - School/Work - Social Relationship - Interests |

**YOUR ADVICE TO OTHER PATIENTS**

**Based upon YOUR experience, would you recommend your appliance to someone who has similar malocclusion?**

| **YES** | **NO** |
| --- | --- |

Supplementary Table 2: Arabic form of validated questionnaire

**تجربتك في ارتداء الجهاز (الطُبيقة) الخاص بك**

| هل لبس الجهاز بالشكل الذي توقعته؟ | **كلا** | **غير متأكد** | **نعم** |
| --- | --- | --- | --- |
| هل تعطل الجهاز خلال فترة العلاج؟ | **أبداً** | **قليلا** | **كثيرا** |
| هل كان لديك أي مواعيد إضافية بسبب تعطل جهازك أو عدم ملاءمته جيداً؟ | **كلا** |  | **نعم** |
| إذا كان لديك مواعيد إضافية بسبب تعطل جهازك أو عدم ملاءمته جيداً، فهل أزعجك ذلك؟ | **أبداً** | **قليلا** | **كثيرا** |
| كان من الصعب الحفاظ على نظافة الجهاز | **أبداً** | **قليلا** | **كثيرا** |
| هل يسقط الجهاز أثناء النوم؟ | **أبداً** | **قليلا** | **كثيرا** |
| كيف تجد إرتداء وإزالة الجهاز؟ | **سهل** |  | **صعب** |
| هل تعاني من أي شعور بالحاجة للتقيؤ أثناء ارتداء الجهاز؟ | **أبداً** | **قليلا** | **كثيرا** |

**كيف تغيرت الأشياء التالية بسبب ارتداء جهازك؟**

| التكلم | تحسن | لم يتغيير | أسوأ |
| --- | --- | --- | --- |
| تناول الطعام | تحسن | لم يتغيير | أسوأ |
| الشرب | تحسن | لم يتغيير | أسوأ |
| تنظيف الأسنان والمحافظة على صحة الفم | تحسن | لم يتغيير | أسوأ |
| التنفس | تحسن | لم يتغيير | أسوأ |
| النوم | تحسن | لم يتغيير | أسوأ |
| الدراسة | تحسن | لم يتغيير | أسوأ |
| المظهر | تحسن | لم تغيير | أسوأ |
| إذا تعرضت للمضايقة أو التنمر بشأن أسنانك قبل بدء العلاج، فهل تغير هذا؟ | أقل | لم يتغيير | **أكثر** |

**كيف أثر عليك ما يلي بسبب ارتداء جهازك؟**

| ألم في أسنانك | أبداً | قليلا | كثيرا |
| --- | --- | --- | --- |
| ألم او تقرح في فمك (بسبب الضغط) | أبداً | قليلا | كثيرا |
| ألم في الفك أو مفاصل الفك | أبداً | قليلا | كثيرا |
| إذا كان هناك ألم، فهل كنت تستخدم الأدوية لتخفيفه؟ | أبداً | قليلا | كثيرا |
| إذا كان هناك ألم، فهل ذلك يؤثر على ارتداء الجهاز | أبداً | قليلا | كثيرا |
| الشعور بالحرج | أبداً | قليلا | كثيرا |
| سيلان اللعاب (بشكل غير مسيطر عليه) | أبداً | قليلا | كثيرا |

**نود معرفة ما إذا كان ارتداء الجهاز يمكن أن يؤثر على أشياء أخرى في حياتك**

**العمل / النشاط المدرسي**

**كيف تأثر عملك / نشاطك المدرسي بسبب ارتداء جهازك؟**

| تحسن | لم يتغيير | أسوأ |
| --- | --- | --- |

**التواصل مع الأصدقاء والعائلة (العلاقات الاجتماعية)**

**كيف تأثر عمل تفاعلك مع الأصدقاء والعائلة بسبب ارتداء جهازك؟**

| تحسن | لم يتغيير | أسوأ |
| --- | --- | --- |

**الهوايات/الاهتمامات**

**إذا كنت تمارس أي هواية، كيف تأثر ذلك بسبب ارتداء جهازك؟**

| تحسن | لم يتغيير | أسوأ |
| --- | --- | --- |

**إذا تحسن أو أصبح أسوأ، فأن ذلك كان بسبب**

| المظهر | تحسن | أسوأ | 🞎 المدرسة/العمل  🞎 العلاقات الاجتماعية  🞎 الاهتمامات |
| --- | --- | --- | --- |
| التكلم | تحسن | أسوأ | 🞎 المدرسة/العمل  🞎 العلاقات الاجتماعية  🞎 الاهتمامات |
| تناول الطعام | تحسن | أسوأ | 🞎 المدرسة/العمل  🞎 العلاقات الاجتماعية |
| الدراسة | تحسن | أسوأ | 🞎 المدرسة/العمل |
| ألم في أسنانك | تحسن | أسوأ | 🞎 المدرسة/العمل  🞎 العلاقات الاجتماعية  🞎 الاهتمامات |
| ألم في فمك أو فكك | تحسن | أسوأ | 🞎 المدرسة/العمل  🞎 العلاقات الاجتماعية  🞎 الاهتمامات |
| الشعور بالحرج | تحسن | أسوأ | 🞎 المدرسة/العمل  🞎 العلاقات الاجتماعية  🞎 الاهتمامات |
| سيلان اللعاب (بشكل غير مسيطر عليه) | تحسن | أسوأ | 🞎 المدرسة/العمل  🞎 العلاقات الاجتماعية  🞎 الاهتمامات |
| المضايقة أو التنمر | تحسن | أسوأ | 🞎 المدرسة/العمل  🞎 العلاقات الاجتماعية  🞎 الاهتمامات |

**نصيحتك للمرضى الآخرين**

**بناءً على تجربتك، هل توصي بجهازك لشخص يعاني من سوء إطباق مماثل؟**

| **نعم** | **كلا** |
| --- | --- |
